# Supplementary material for: Modeling glioblastoma heterogeneity as a dynamic network of cell states
Source: Mol Syst Biol. 2021 Sep 16;17(9):e10105. doi: 10.15252/msb.202010105 (PMC8444284; doi:10.15252/msb.202010105)
Supplement: Supplementary file 5 — Source Data for Figure 3 [file MSB-17-e10105-s001.zip › Figure3A_sourcedata/GSEA_3065/nosigdb_state3.GseaPreranked.1622718426028/VANGRONINGEN_MES.html]

Details for gene set VANGRONINGEN\_MES[GSEA]

|  || Dataset | state3 |
| Phenotype | NoPhenotypeAvailable |
| Upregulated in class | na\_pos |
| GeneSet | VANGRONINGEN\_MES |
| Enrichment Score (ES) | 0.5102799 |
| Normalized Enrichment Score (NES) | 2.3443274 |
| Nominal p-value | 0.0 |
| FDR q-value | 0.0 |
| FWER p-Value | 0.0 |
Table: GSEA Results Summary

  

Fig 1: Enrichment plot: VANGRONINGEN\_MES      
 Profile of the Running ES Score & Positions of GeneSet Members on the Rank Ordered List

  

| PROBE | GENE SYMBOL | GENE\_TITLE | RANK IN GENE LIST | RANK METRIC SCORE | RUNNING ES | CORE ENRICHMENT || 1 | SCRG1 |  |  | 3 | 0.645 | 0.0120 | Yes |
| 2 | HSP90B1 |  |  | 7 | 0.563 | 0.0224 | Yes |
| 3 | PDIA6 |  |  | 8 | 0.549 | 0.0329 | Yes |
| 4 | B2M |  |  | 11 | 0.538 | 0.0430 | Yes |
| 5 | LMAN1 |  |  | 21 | 0.496 | 0.0514 | Yes |
| 6 | PTN |  |  | 22 | 0.493 | 0.0609 | Yes |
| 7 | POSTN |  |  | 24 | 0.487 | 0.0701 | Yes |
| 8 | CD63 |  |  | 26 | 0.480 | 0.0792 | Yes |
| 9 | TIMP1 |  |  | 35 | 0.470 | 0.0872 | Yes |
| 10 | PRDX4 |  |  | 41 | 0.455 | 0.0953 | Yes |
| 11 | HSPA5 |  |  | 43 | 0.447 | 0.1038 | Yes |
| 12 | CALU |  |  | 48 | 0.437 | 0.1117 | Yes |
| 13 | APP |  |  | 49 | 0.437 | 0.1200 | Yes |
| 14 | PDIA3 |  |  | 55 | 0.419 | 0.1274 | Yes |
| 15 | WLS |  |  | 58 | 0.407 | 0.1350 | Yes |
| 16 | PON2 |  |  | 63 | 0.389 | 0.1420 | Yes |
| 17 | MANF |  |  | 70 | 0.376 | 0.1485 | Yes |
| 18 | TMED9 |  |  | 75 | 0.370 | 0.1551 | Yes |
| 19 | SPCS3 |  |  | 76 | 0.370 | 0.1622 | Yes |
| 20 | ITM2B |  |  | 82 | 0.361 | 0.1685 | Yes |
| 21 | FAT1 |  |  | 95 | 0.350 | 0.1738 | Yes |
| 22 | LAMC1 |  |  | 96 | 0.349 | 0.1804 | Yes |
| 23 | DDOST |  |  | 106 | 0.343 | 0.1859 | Yes |
| 24 | NES |  |  | 113 | 0.332 | 0.1916 | Yes |
| 25 | OSTC |  |  | 118 | 0.325 | 0.1973 | Yes |
| 26 | PDIA4 |  |  | 120 | 0.324 | 0.2034 | Yes |
| 27 | CALD1 |  |  | 124 | 0.322 | 0.2092 | Yes |
| 28 | TJP1 |  |  | 139 | 0.314 | 0.2136 | Yes |
| 29 | PPIB |  |  | 142 | 0.313 | 0.2193 | Yes |
| 30 | ITGB1 |  |  | 150 | 0.310 | 0.2244 | Yes |
| 31 | PRCP |  |  | 166 | 0.303 | 0.2284 | Yes |
| 32 | DNAJC3 |  |  | 182 | 0.294 | 0.2323 | Yes |
| 33 | FKBP14 |  |  | 185 | 0.292 | 0.2376 | Yes |
| 34 | TXNDC12 |  |  | 186 | 0.292 | 0.2432 | Yes |
| 35 | TM9SF2 |  |  | 189 | 0.291 | 0.2486 | Yes |
| 36 | TMBIM4 |  |  | 204 | 0.281 | 0.2523 | Yes |
| 37 | LAPTM4A |  |  | 208 | 0.278 | 0.2572 | Yes |
| 38 | RRBP1 |  |  | 220 | 0.274 | 0.2612 | Yes |
| 39 | CTSC |  |  | 226 | 0.272 | 0.2658 | Yes |
| 40 | DNAJC1 |  |  | 227 | 0.272 | 0.2710 | Yes |
| 41 | ADAM9 |  |  | 229 | 0.272 | 0.2761 | Yes |
| 42 | COL4A2 |  |  | 232 | 0.271 | 0.2810 | Yes |
| 43 | RCN1 |  |  | 233 | 0.270 | 0.2862 | Yes |
| 44 | KDELR2 |  |  | 238 | 0.269 | 0.2909 | Yes |
| 45 | COL4A1 |  |  | 241 | 0.267 | 0.2957 | Yes |
| 46 | TNC |  |  | 249 | 0.264 | 0.3000 | Yes |
| 47 | TMEM50A |  |  | 253 | 0.262 | 0.3046 | Yes |
| 48 | COL6A1 |  |  | 254 | 0.261 | 0.3096 | Yes |
| 49 | CMTM6 |  |  | 255 | 0.261 | 0.3146 | Yes |
| 50 | SYPL1 |  |  | 261 | 0.258 | 0.3190 | Yes |
| 51 | EPHA3 |  |  | 262 | 0.257 | 0.3239 | Yes |
| 52 | SSR3 |  |  | 264 | 0.255 | 0.3287 | Yes |
| 53 | COL5A2 |  |  | 266 | 0.255 | 0.3334 | Yes |
| 54 | PTPRG |  |  | 268 | 0.254 | 0.3382 | Yes |
| 55 | ATP2B1 |  |  | 271 | 0.253 | 0.3428 | Yes |
| 56 | ITM2C |  |  | 273 | 0.252 | 0.3475 | Yes |
| 57 | LAMP1 |  |  | 287 | 0.248 | 0.3507 | Yes |
| 58 | CD164 |  |  | 288 | 0.247 | 0.3554 | Yes |
| 59 | PHTF2 |  |  | 292 | 0.245 | 0.3597 | Yes |
| 60 | HEXB |  |  | 298 | 0.244 | 0.3638 | Yes |
| 61 | COL5A1 |  |  | 302 | 0.242 | 0.3681 | Yes |
| 62 | SERPINE2 |  |  | 308 | 0.241 | 0.3721 | Yes |
| 63 | SURF4 |  |  | 311 | 0.240 | 0.3765 | Yes |
| 64 | IL6ST |  |  | 316 | 0.239 | 0.3806 | Yes |
| 65 | JAM3 |  |  | 317 | 0.239 | 0.3851 | Yes |
| 66 | ITGAV |  |  | 322 | 0.238 | 0.3892 | Yes |
| 67 | MYDGF |  |  | 329 | 0.234 | 0.3930 | Yes |
| 68 | ASPH |  |  | 332 | 0.234 | 0.3972 | Yes |
| 69 | IGF2R |  |  | 348 | 0.230 | 0.3998 | Yes |
| 70 | FUCA2 |  |  | 351 | 0.229 | 0.4040 | Yes |
| 71 | NPTN |  |  | 355 | 0.229 | 0.4080 | Yes |
| 72 | SPARC |  |  | 361 | 0.228 | 0.4118 | Yes |
| 73 | PXDN |  |  | 362 | 0.227 | 0.4161 | Yes |
| 74 | TNFRSF12A |  |  | 380 | 0.223 | 0.4184 | Yes |
| 75 | CTSB |  |  | 392 | 0.219 | 0.4213 | Yes |
| 76 | MAGT1 |  |  | 394 | 0.219 | 0.4254 | Yes |
| 77 | FAM3C |  |  | 406 | 0.217 | 0.4282 | Yes |
| 78 | LAMB1 |  |  | 415 | 0.215 | 0.4314 | Yes |
| 79 | FGFR1 |  |  | 419 | 0.215 | 0.4351 | Yes |
| 80 | PLEKHH2 |  |  | 440 | 0.211 | 0.4368 | Yes |
| 81 | PLOD3 |  |  | 443 | 0.210 | 0.4406 | Yes |
| 82 | GPC6 |  |  | 468 | 0.204 | 0.4416 | Yes |
| 83 | TFPI |  |  | 480 | 0.202 | 0.4442 | Yes |
| 84 | TRAM1 |  |  | 519 | 0.196 | 0.4434 | Yes |
| 85 | ADGRG6 |  |  | 526 | 0.195 | 0.4464 | Yes |
| 86 | GRN |  |  | 551 | 0.192 | 0.4472 | Yes |
| 87 | DNAJC10 |  |  | 559 | 0.191 | 0.4501 | Yes |
| 88 | TRAM2 |  |  | 569 | 0.190 | 0.4526 | Yes |
| 89 | A2M |  |  | 585 | 0.188 | 0.4545 | Yes |
| 90 | SERPINH1 |  |  | 588 | 0.188 | 0.4578 | Yes |
| 91 | FLNA |  |  | 633 | 0.181 | 0.4560 | Yes |
| 92 | EXTL2 |  |  | 637 | 0.181 | 0.4591 | Yes |
| 93 | ROBO1 |  |  | 644 | 0.179 | 0.4619 | Yes |
| 94 | P4HA1 |  |  | 649 | 0.179 | 0.4648 | Yes |
| 95 | CRELD2 |  |  | 655 | 0.177 | 0.4676 | Yes |
| 96 | PPT1 |  |  | 657 | 0.177 | 0.4709 | Yes |
| 97 | PLOD2 |  |  | 683 | 0.173 | 0.4712 | Yes |
| 98 | COL11A1 |  |  | 684 | 0.173 | 0.4745 | Yes |
| 99 | CD59 |  |  | 713 | 0.168 | 0.4744 | Yes |
| 100 | FBN1 |  |  | 768 | 0.161 | 0.4711 | Yes |
| 101 | FSTL1 |  |  | 790 | 0.159 | 0.4716 | Yes |
| 102 | KDELC2 |  |  | 795 | 0.158 | 0.4741 | Yes |
| 103 | LOXL2 |  |  | 817 | 0.156 | 0.4746 | Yes |
| 104 | VCL |  |  | 834 | 0.154 | 0.4757 | Yes |
| 105 | CRISPLD1 |  |  | 840 | 0.154 | 0.4780 | Yes |
| 106 | SSR1 |  |  | 851 | 0.152 | 0.4797 | Yes |
| 107 | COPA |  |  | 853 | 0.152 | 0.4825 | Yes |
| 108 | CYR61 |  |  | 873 | 0.150 | 0.4831 | Yes |
| 109 | GNS |  |  | 875 | 0.150 | 0.4859 | Yes |
| 110 | TWSG1 |  |  | 893 | 0.149 | 0.4867 | Yes |
| 111 | HYOU1 |  |  | 906 | 0.148 | 0.4881 | Yes |
| 112 | IQGAP2 |  |  | 943 | 0.143 | 0.4866 | Yes |
| 113 | TM4SF1 |  |  | 952 | 0.142 | 0.4883 | Yes |
| 114 | HTRA1 |  |  | 955 | 0.142 | 0.4908 | Yes |
| 115 | STAT3 |  |  | 965 | 0.141 | 0.4924 | Yes |
| 116 | CCDC80 |  |  | 966 | 0.141 | 0.4951 | Yes |
| 117 | FN1 |  |  | 973 | 0.140 | 0.4971 | Yes |
| 118 | LEPROT |  |  | 974 | 0.140 | 0.4998 | Yes |
| 119 | PTGFRN |  |  | 979 | 0.140 | 0.5020 | Yes |
| 120 | SCPEP1 |  |  | 1065 | 0.133 | 0.4944 | Yes |
| 121 | ACTN1 |  |  | 1079 | 0.132 | 0.4954 | Yes |
| 122 | ATP1B1 |  |  | 1102 | 0.129 | 0.4953 | Yes |
| 123 | GPX8 |  |  | 1138 | 0.127 | 0.4935 | Yes |
| 124 | OLFML2A |  |  | 1150 | 0.126 | 0.4946 | Yes |
| 125 | GJA1 |  |  | 1160 | 0.125 | 0.4959 | Yes |
| 126 | MRC2 |  |  | 1162 | 0.125 | 0.4982 | Yes |
| 127 | ANTXR1 |  |  | 1180 | 0.123 | 0.4985 | Yes |
| 128 | CRTAP |  |  | 1189 | 0.122 | 0.4999 | Yes |
| 129 | ERLIN1 |  |  | 1190 | 0.122 | 0.5023 | Yes |
| 130 | PTPRK |  |  | 1207 | 0.121 | 0.5027 | Yes |
| 131 | SLC30A7 |  |  | 1208 | 0.121 | 0.5050 | Yes |
| 132 | RNFT1 |  |  | 1223 | 0.120 | 0.5056 | Yes |
| 133 | CYFIP1 |  |  | 1224 | 0.120 | 0.5079 | Yes |
| 134 | LIFR |  |  | 1260 | 0.117 | 0.5060 | Yes |
| 135 | CDH11 |  |  | 1289 | 0.116 | 0.5049 | Yes |
| 136 | SLC35F5 |  |  | 1302 | 0.115 | 0.5056 | Yes |
| 137 | F2R |  |  | 1308 | 0.115 | 0.5072 | Yes |
| 138 | GDF15 |  |  | 1318 | 0.114 | 0.5083 | Yes |
| 139 | SLC38A2 |  |  | 1321 | 0.114 | 0.5103 | Yes |
| 140 | DPY19L1 |  |  | 1341 | 0.112 | 0.5102 | No |
| 141 | MBTPS1 |  |  | 1379 | 0.110 | 0.5079 | No |
| 142 | GALNT10 |  |  | 1380 | 0.110 | 0.5100 | No |
| 143 | WNT5A |  |  | 1428 | 0.107 | 0.5064 | No |
| 144 | DDR2 |  |  | 1443 | 0.106 | 0.5068 | No |
| 145 | SPRY4 |  |  | 1461 | 0.105 | 0.5067 | No |
| 146 | SPRY1 |  |  | 1471 | 0.105 | 0.5077 | No |
| 147 | SEMA3C |  |  | 1479 | 0.105 | 0.5088 | No |
| 148 | HES1 |  |  | 1501 | 0.103 | 0.5083 | No |
| 149 | DKK3 |  |  | 1538 | 0.101 | 0.5059 | No |
| 150 | NID1 |  |  | 1545 | 0.101 | 0.5072 | No |
| 151 | THBS1 |  |  | 1561 | 0.100 | 0.5073 | No |
| 152 | PYGL |  |  | 1570 | 0.099 | 0.5082 | No |
| 153 | IFI16 |  |  | 1576 | 0.099 | 0.5095 | No |
| 154 | TOR1AIP1 |  |  | 1598 | 0.098 | 0.5089 | No |
| 155 | EMP1 |  |  | 1624 | 0.096 | 0.5078 | No |
| 156 | KDELR3 |  |  | 1678 | 0.093 | 0.5032 | No |
| 157 | CREB3L2 |  |  | 1691 | 0.092 | 0.5036 | No |
| 158 | LPP |  |  | 1725 | 0.090 | 0.5014 | No |
| 159 | CKAP4 |  |  | 1727 | 0.090 | 0.5030 | No |
| 160 | CD44 |  |  | 1748 | 0.089 | 0.5023 | No |
| 161 | FZD2 |  |  | 1774 | 0.088 | 0.5010 | No |
| 162 | MYADM |  |  | 1797 | 0.087 | 0.5000 | No |
| 163 | NPC2 |  |  | 1842 | 0.084 | 0.4964 | No |
| 164 | SDC2 |  |  | 1875 | 0.082 | 0.4941 | No |
| 165 | MAML2 |  |  | 1883 | 0.082 | 0.4949 | No |
| 166 | SLC39A14 |  |  | 1886 | 0.082 | 0.4962 | No |
| 167 | MMP2 |  |  | 1898 | 0.081 | 0.4964 | No |
| 168 | MGAT2 |  |  | 1906 | 0.081 | 0.4971 | No |
| 169 | LUZP1 |  |  | 1915 | 0.080 | 0.4977 | No |
| 170 | SFT2D2 |  |  | 1976 | 0.077 | 0.4920 | No |
| 171 | PEAK1 |  |  | 1984 | 0.077 | 0.4927 | No |
| 172 | NOTCH2 |  |  | 1990 | 0.076 | 0.4935 | No |
| 173 | ANXA6 |  |  | 2013 | 0.075 | 0.4924 | No |
| 174 | TSPAN4 |  |  | 2018 | 0.075 | 0.4933 | No |
| 175 | SDF4 |  |  | 2052 | 0.074 | 0.4908 | No |
| 176 | DSE |  |  | 2105 | 0.072 | 0.4860 | No |
| 177 | ARHGEF40 |  |  | 2118 | 0.071 | 0.4859 | No |
| 178 | PPIC |  |  | 2162 | 0.070 | 0.4821 | No |
| 179 | SDC4 |  |  | 2189 | 0.069 | 0.4803 | No |
| 180 | LRRC17 |  |  | 2223 | 0.067 | 0.4777 | No |
| 181 | AEBP1 |  |  | 2257 | 0.066 | 0.4750 | No |
| 182 | RECK |  |  | 2272 | 0.065 | 0.4746 | No |
| 183 | QSOX1 |  |  | 2301 | 0.064 | 0.4725 | No |
| 184 | MAN2A1 |  |  | 2313 | 0.064 | 0.4724 | No |
| 185 | CYBRD1 |  |  | 2317 | 0.064 | 0.4733 | No |
| 186 | SLC16A4 |  |  | 2321 | 0.064 | 0.4741 | No |
| 187 | ADGRE5 |  |  | 2356 | 0.063 | 0.4713 | No |
| 188 | PTBP1 |  |  | 2359 | 0.062 | 0.4722 | No |
| 189 | KDM5B |  |  | 2370 | 0.062 | 0.4722 | No |
| 190 | EGFR |  |  | 2416 | 0.060 | 0.4680 | No |
| 191 | SEPT10 |  |  | 2425 | 0.060 | 0.4682 | No |
| 192 | LRP10 |  |  | 2436 | 0.059 | 0.4681 | No |
| 193 | ITPRIPL2 |  |  | 2455 | 0.059 | 0.4671 | No |
| 194 | ATP2B4 |  |  | 2510 | 0.057 | 0.4618 | No |
| 195 | GPR137B |  |  | 2521 | 0.056 | 0.4616 | No |
| 196 | COL6A2 |  |  | 2565 | 0.055 | 0.4576 | No |
| 197 | ENAH |  |  | 2572 | 0.054 | 0.4579 | No |
| 198 | IL13RA1 |  |  | 2577 | 0.054 | 0.4584 | No |
| 199 | TSC22D2 |  |  | 2604 | 0.053 | 0.4564 | No |
| 200 | PTPN14 |  |  | 2681 | 0.051 | 0.4483 | No |
| 201 | ERBIN |  |  | 2684 | 0.050 | 0.4490 | No |
| 202 | TNFRSF1A |  |  | 2695 | 0.050 | 0.4488 | No |
| 203 | EDEM1 |  |  | 2724 | 0.049 | 0.4464 | No |
| 204 | UGDH |  |  | 2879 | 0.045 | 0.4289 | No |
| 205 | LIPA |  |  | 2893 | 0.044 | 0.4282 | No |
| 206 | SEL1L3 |  |  | 2895 | 0.044 | 0.4289 | No |
| 207 | CTSO |  |  | 2920 | 0.043 | 0.4269 | No |
| 208 | AMMECR1 |  |  | 2934 | 0.043 | 0.4261 | No |
| 209 | ELK4 |  |  | 2962 | 0.042 | 0.4237 | No |
| 210 | CLIC4 |  |  | 2993 | 0.041 | 0.4209 | No |
| 211 | SFT2D1 |  |  | 3004 | 0.041 | 0.4205 | No |
| 212 | PXDC1 |  |  | 3039 | 0.040 | 0.4172 | No |
| 213 | FNDC3B |  |  | 3115 | 0.038 | 0.4090 | No |
| 214 | ELAVL1 |  |  | 3125 | 0.038 | 0.4087 | No |
| 215 | INSIG1 |  |  | 3136 | 0.038 | 0.4082 | No |
| 216 | PDGFC |  |  | 3169 | 0.037 | 0.4051 | No |
| 217 | ARMCX2 |  |  | 3295 | 0.033 | 0.3908 | No |
| 218 | CBFB |  |  | 3344 | 0.032 | 0.3857 | No |
| 219 | RGL1 |  |  | 3374 | 0.031 | 0.3828 | No |
| 220 | IFITM3 |  |  | 3449 | 0.029 | 0.3746 | No |
| 221 | ATXN1 |  |  | 3504 | 0.028 | 0.3687 | No |
| 222 | SRPX |  |  | 3518 | 0.028 | 0.3676 | No |
| 223 | IGFBP5 |  |  | 3555 | 0.027 | 0.3639 | No |
| 224 | STK38L |  |  | 3574 | 0.026 | 0.3622 | No |
| 225 | ACADVL |  |  | 3600 | 0.026 | 0.3597 | No |
| 226 | ELK3 |  |  | 3671 | 0.024 | 0.3518 | No |
| 227 | ADAM19 |  |  | 3674 | 0.024 | 0.3520 | No |
| 228 | ATP10D |  |  | 3714 | 0.022 | 0.3478 | No |
| 229 | C6orf120 |  |  | 3724 | 0.022 | 0.3472 | No |
| 230 | KANK2 |  |  | 3747 | 0.021 | 0.3450 | No |
| 231 | SMAD3 |  |  | 3767 | 0.021 | 0.3431 | No |
| 232 | TGFBR2 |  |  | 3803 | 0.020 | 0.3393 | No |
| 233 | CTNNA1 |  |  | 3806 | 0.020 | 0.3394 | No |
| 234 | OGFRL1 |  |  | 3920 | 0.017 | 0.3263 | No |
| 235 | SPRED1 |  |  | 3989 | 0.016 | 0.3185 | No |
| 236 | PLS3 |  |  | 4021 | 0.015 | 0.3151 | No |
| 237 | GSN |  |  | 4064 | 0.013 | 0.3103 | No |
| 238 | TRIM5 |  |  | 4097 | 0.013 | 0.3067 | No |
| 239 | EGR1 |  |  | 4134 | 0.012 | 0.3027 | No |
| 240 | GAS1 |  |  | 4210 | 0.009 | 0.2939 | No |
| 241 | REST |  |  | 4235 | 0.009 | 0.2912 | No |
| 242 | RAB29 |  |  | 4279 | 0.007 | 0.2862 | No |
| 243 | LASP1 |  |  | 4284 | 0.007 | 0.2859 | No |
| 244 | PLSCR1 |  |  | 4373 | 0.005 | 0.2755 | No |
| 245 | ELF1 |  |  | 4377 | 0.004 | 0.2752 | No |
| 246 | FAM129A |  |  | 4493 | 0.001 | 0.2615 | No |
| 247 | SOX9 |  |  | 4503 | 0.001 | 0.2605 | No |
| 248 | SHC1 |  |  | 4506 | 0.001 | 0.2602 | No |
| 249 | ARL1 |  |  | 4539 | 0.000 | 0.2564 | No |
| 250 | STAT1 |  |  | 4591 | -0.001 | 0.2504 | No |
| 251 | SASH1 |  |  | 4645 | -0.002 | 0.2441 | No |
| 252 | KIF13A |  |  | 4653 | -0.003 | 0.2433 | No |
| 253 | TMEFF2 |  |  | 4708 | -0.004 | 0.2369 | No |
| 254 | PLPP1 |  |  | 4721 | -0.004 | 0.2356 | No |
| 255 | ZCCHC24 |  |  | 4731 | -0.004 | 0.2346 | No |
| 256 | FIBIN |  |  | 4788 | -0.006 | 0.2280 | No |
| 257 | EPS8 |  |  | 4819 | -0.007 | 0.2246 | No |
| 258 | ZNF217 |  |  | 4854 | -0.007 | 0.2207 | No |
| 259 | HIPK3 |  |  | 4901 | -0.008 | 0.2153 | No |
| 260 | SHROOM3 |  |  | 4902 | -0.008 | 0.2155 | No |
| 261 | PHLDB2 |  |  | 4997 | -0.011 | 0.2045 | No |
| 262 | TPBG |  |  | 5003 | -0.011 | 0.2041 | No |
| 263 | YAP1 |  |  | 5041 | -0.012 | 0.1999 | No |
| 264 | SKIL |  |  | 5063 | -0.012 | 0.1977 | No |
| 265 | IFITM2 |  |  | 5106 | -0.014 | 0.1929 | No |
| 266 | FZD7 |  |  | 5109 | -0.014 | 0.1929 | No |
| 267 | SPARCL1 |  |  | 5152 | -0.014 | 0.1882 | No |
| 268 | CXCL12 |  |  | 5164 | -0.015 | 0.1872 | No |
| 269 | HOMER1 |  |  | 5174 | -0.015 | 0.1864 | No |
| 270 | L3HYPDH |  |  | 5182 | -0.015 | 0.1859 | No |
| 271 | GNAI1 |  |  | 5194 | -0.016 | 0.1849 | No |
| 272 | CTDSP2 |  |  | 5249 | -0.017 | 0.1787 | No |
| 273 | DMD |  |  | 5320 | -0.019 | 0.1707 | No |
| 274 | EHD2 |  |  | 5399 | -0.021 | 0.1618 | No |
| 275 | SNAP23 |  |  | 5441 | -0.022 | 0.1574 | No |
| 276 | RIT1 |  |  | 5453 | -0.023 | 0.1565 | No |
| 277 | HS3ST3A1 |  |  | 5482 | -0.024 | 0.1536 | No |
| 278 | KLF10 |  |  | 5514 | -0.025 | 0.1504 | No |
| 279 | ACAP2 |  |  | 5531 | -0.025 | 0.1490 | No |
| 280 | KLF6 |  |  | 5627 | -0.028 | 0.1382 | No |
| 281 | TPM1 |  |  | 5672 | -0.029 | 0.1335 | No |
| 282 | NR3C1 |  |  | 5724 | -0.030 | 0.1280 | No |
| 283 | C1orf198 |  |  | 5750 | -0.031 | 0.1256 | No |
| 284 | ARHGAP1 |  |  | 5751 | -0.031 | 0.1262 | No |
| 285 | TPM2 |  |  | 5823 | -0.034 | 0.1184 | No |
| 286 | SYNJ2 |  |  | 5836 | -0.034 | 0.1176 | No |
| 287 | UAP1 |  |  | 5859 | -0.035 | 0.1156 | No |
| 288 | RHOJ |  |  | 5872 | -0.035 | 0.1149 | No |
| 289 | PLEKHA2 |  |  | 5902 | -0.036 | 0.1121 | No |
| 290 | FAM114A1 |  |  | 5903 | -0.036 | 0.1128 | No |
| 291 | TCF7L2 |  |  | 5935 | -0.037 | 0.1098 | No |
| 292 | MBNL1 |  |  | 6042 | -0.041 | 0.0979 | No |
| 293 | SVIL |  |  | 6089 | -0.042 | 0.0932 | No |
| 294 | RBMS1 |  |  | 6094 | -0.043 | 0.0936 | No |
| 295 | JAK1 |  |  | 6114 | -0.043 | 0.0922 | No |
| 296 | LIX1L |  |  | 6184 | -0.046 | 0.0848 | No |
| 297 | NBR1 |  |  | 6233 | -0.047 | 0.0800 | No |
| 298 | SEC14L1 |  |  | 6272 | -0.049 | 0.0764 | No |
| 299 | BAG3 |  |  | 6417 | -0.053 | 0.0602 | No |
| 300 | SUCLG2 |  |  | 6466 | -0.055 | 0.0556 | No |
| 301 | MEST |  |  | 6477 | -0.056 | 0.0554 | No |
| 302 | TFE3 |  |  | 6478 | -0.056 | 0.0565 | No |
| 303 | WIPI1 |  |  | 6485 | -0.056 | 0.0569 | No |
| 304 | DUSP6 |  |  | 6502 | -0.056 | 0.0560 | No |
| 305 | RNH1 |  |  | 6509 | -0.057 | 0.0564 | No |
| 306 | QKI |  |  | 6517 | -0.057 | 0.0567 | No |
| 307 | ANXA5 |  |  | 6522 | -0.057 | 0.0573 | No |
| 308 | NFIC |  |  | 6539 | -0.058 | 0.0565 | No |
| 309 | DCAF6 |  |  | 6578 | -0.060 | 0.0531 | No |
| 310 | ERRFI1 |  |  | 6678 | -0.065 | 0.0425 | No |
| 311 | PLK2 |  |  | 6726 | -0.067 | 0.0382 | No |
| 312 | FILIP1L |  |  | 6892 | -0.075 | 0.0200 | No |
| 313 | ZFP36L1 |  |  | 6893 | -0.076 | 0.0214 | No |
| 314 | CSRP1 |  |  | 6916 | -0.077 | 0.0203 | No |
| 315 | PALLD |  |  | 6929 | -0.077 | 0.0203 | No |
| 316 | CMTM3 |  |  | 6951 | -0.078 | 0.0193 | No |
| 317 | PDLIM1 |  |  | 6965 | -0.079 | 0.0193 | No |
| 318 | ANXA1 |  |  | 7032 | -0.083 | 0.0130 | No |
| 319 | ATP6V0E1 |  |  | 7106 | -0.088 | 0.0060 | No |
| 320 | NANS |  |  | 7169 | -0.092 | 0.0003 | No |
| 321 | BOC |  |  | 7182 | -0.092 | 0.0007 | No |
| 322 | WWTR1 |  |  | 7187 | -0.093 | 0.0020 | No |
| 323 | SPATA20 |  |  | 7228 | -0.095 | -0.0010 | No |
| 324 | RGS3 |  |  | 7262 | -0.097 | -0.0031 | No |
| 325 | DESI2 |  |  | 7284 | -0.099 | -0.0037 | No |
| 326 | SSBP4 |  |  | 7369 | -0.106 | -0.0117 | No |
| 327 | TUBB6 |  |  | 7395 | -0.108 | -0.0126 | No |
| 328 | LMNA |  |  | 7412 | -0.109 | -0.0124 | No |
| 329 | CETN2 |  |  | 7440 | -0.112 | -0.0135 | No |
| 330 | SDCBP |  |  | 7454 | -0.113 | -0.0129 | No |
| 331 | EFEMP2 |  |  | 7457 | -0.113 | -0.0109 | No |
| 332 | TGFB1I1 |  |  | 7478 | -0.115 | -0.0111 | No |
| 333 | MYL12A |  |  | 7482 | -0.115 | -0.0093 | No |
| 334 | MOB1A |  |  | 7518 | -0.119 | -0.0112 | No |
| 335 | HIBADH |  |  | 7523 | -0.119 | -0.0094 | No |
| 336 | RAB13 |  |  | 7553 | -0.122 | -0.0105 | No |
| 337 | FAM120A |  |  | 7624 | -0.130 | -0.0164 | No |
| 338 | PRRX1 |  |  | 7670 | -0.136 | -0.0191 | No |
| 339 | SYDE1 |  |  | 7711 | -0.141 | -0.0212 | No |
| 340 | DUSP14 |  |  | 7727 | -0.143 | -0.0203 | No |
| 341 | ARL4A |  |  | 7829 | -0.155 | -0.0293 | No |
| 342 | PHLDA3 |  |  | 7839 | -0.157 | -0.0274 | No |
| 343 | RGS10 |  |  | 7850 | -0.158 | -0.0256 | No |
| 344 | MBD2 |  |  | 7865 | -0.160 | -0.0242 | No |
| 345 | CAPN2 |  |  | 7878 | -0.162 | -0.0225 | No |
| 346 | ID1 |  |  | 7938 | -0.175 | -0.0262 | No |
| 347 | RAP1B |  |  | 7964 | -0.179 | -0.0257 | No |
| 348 | SH3BGRL |  |  | 7984 | -0.182 | -0.0245 | No |
| 349 | TMEM263 |  |  | 7985 | -0.183 | -0.0210 | No |
| 350 | LITAF |  |  | 8015 | -0.188 | -0.0209 | No |
| 351 | ABRACL |  |  | 8121 | -0.209 | -0.0294 | No |
| 352 | ARPC1B |  |  | 8131 | -0.212 | -0.0264 | No |
| 353 | SQSTM1 |  |  | 8176 | -0.221 | -0.0274 | No |
| 354 | RAP1A |  |  | 8198 | -0.228 | -0.0256 | No |
| 355 | ANXA2 |  |  | 8206 | -0.230 | -0.0220 | No |
| 356 | CNN3 |  |  | 8228 | -0.234 | -0.0200 | No |
| 357 | NQO1 |  |  | 8263 | -0.244 | -0.0194 | No |
| 358 | MYL12B |  |  | 8269 | -0.246 | -0.0153 | No |
| 359 | GNG12 |  |  | 8323 | -0.264 | -0.0166 | No |
| 360 | PEA15 |  |  | 8347 | -0.272 | -0.0141 | No |
| 361 | PRDX6 |  |  | 8410 | -0.300 | -0.0158 | No |
| 362 | ID3 |  |  | 8502 | -0.341 | -0.0201 | No |
| 363 | HSPB1 |  |  | 8572 | -0.383 | -0.0210 | No |
| 364 | POLR2L |  |  | 8664 | -0.468 | -0.0229 | No |
| 365 | RHOC |  |  | 8675 | -0.484 | -0.0148 | No |
| 366 | RAB31 |  |  | 8696 | -0.514 | -0.0073 | No |
| 367 | LGALS1 |  |  | 8744 | -0.718 | 0.0008 | No |
Table: GSEA details [plain text format]

  

Fig 2: VANGRONINGEN\_MES: Random ES distribution      
 Gene set null distribution of ES for **VANGRONINGEN\_MES**
